# A Case Series on Enteroviral Meningitis in Pakistan

**DOI:** 10.7759/cureus.19048

**Published:** 2021-10-26

**Authors:** Lubna Jafri, Ayisha Farooq Khan, Tooba Arshad, Dureshahwar Kanwar

**Affiliations:** 1 Neurology, Aga Khan University Hospital, Karachi, PAK

**Keywords:** headache, case series, meningitis, viral meningitis, enterovirus

## Abstract

Enteroviruses (EVs) are the most common cause of viral meningitis with a peak incidence between late summer and fall. The onset of symptoms is characteristically abrupt and typically includes headache, fever, nausea or vomiting, malaise, photophobia, and meningismus. In addition, diarrhea, upper respiratory symptoms, and a rash may also be present. The clinical presentation and epidemiologic features help in the diagnosis and it is confirmed by the detection of RNA in the cerebrospinal fluid (CSF) by polymerase chain reaction (PCR). We present the clinical description, diagnosis, and management of five consecutive cases of viral meningitis secondary to enterovirus that presented to the emergency department at a tertiary care center in Karachi, Pakistan, over a span of five weeks during the monsoon season. These cases or outbreaks have not been reported previously in Pakistan and, hence, this case series is the first of its kind.

All of our patients were young males, with ages between 18-35 years, did not have any prior co-morbidities, and resided in different localities of Karachi, Pakistan. The presenting complaints were severe headache in all five patients (100%), fever in all five patients (100%), and diarrhea in two out of five patients (40%). On examination, neck stiffness was present in all patients (100%). After the required workup and detection of RNA in the CSF by PCR, diagnosis of enteroviral meningitis was confirmed. The patients were given symptomatic treatment and discharged home with no neurologic complications. Aseptic meningitis occurring during the summer or fall is most likely to be caused by non-polio EVs (eg. coxsackievirus, echovirus, etc.). It is self-limiting and only requires supportive treatment. However, clinically it cannot be differentiated from other central nervous system infections and significant morbidity has been reported, including hospitalization and impairment of routine activities.

## Introduction

Enteroviruses (EVs) are the most common cause of viral meningitis in all ages [1]. An EV is a single-stranded RNA virus that is part of the family of Picornaviridae which comprises 10 species of true EVs and three species of rhinoviruses [2]. Amongst these, coxsackieviruses and echoviruses are the major causes of aseptic meningitis worldwide. There are different serotypes, each accounting for a different infection. Treatment is supportive and there is a good prognosis. Therefore, it is important to establish the diagnosis in order to decrease the unnecessary use of antibiotics. Enteroviral meningitis is a well-known entity; however, it is seldom diagnosed and reported in developing countries such as Pakistan. Lack of laboratory facilities and financial constraints are the major contributing factors. Here we present a case series of five patients with enteroviral meningitis and subsequently a rapid review on the same. To the best of our knowledge, this is the first set of cases of enteroviral meningitis reported in Pakistan. 

## Case presentation

Case 1

A 25-year-old male with no prior co-morbidities presented to the ED in August 2020 with fever, vomiting, and loose stools for four days. Fever was high grade, intermittent, associated with chills, and relieved by taking oral antipyretics. The headache had a sudden onset, was severe, throbbing in nature, involving the bilateral frontal region, and associated with photophobia and phonophobia. There was no blurring of vision, weakness, numbness, altered mentation, seizures, or loss of consciousness.

On arrival to the ED, he was awake, alert, and well-oriented to time, place, and person. He was afebrile with a BP of 108/66 mmHg and heart rate of 66 beats per minute. Pupils were bilaterally equal, round, and reactive to light. Neck stiffness was present. Cranial nerves examination was unremarkable. Motor and sensory systems were normal. There was generalized abdominal tenderness. Other systemic examinations were within normal limits. A clinical diagnosis of meningitis was made, which was confirmed by an elevated total leukocyte count with 84% lymphocytes on cerebrospinal fluid (CSF) studies. EV PCR was detected on biofire filmarray meningitis/encephalitis panel (BFM) in the CSF. MRI brain with contrast was unremarkable. Results of other laboratory investigations are as shown in Table 1.

**Table 1:** Laboratory parameters for the five cases Hb (hemoglobin): normal range: 13.5 to 17.5 g/dL (males) and 12.0 to 15.5 g/dL (females); WBC (white blood cell count): normal range: 3.4–9.06 × 10^9^/L; Platelet count: normal range: 165–415 × 10^9^/L; AST (aspartate aminotransferase): normal range: 12–38IU/L; ALT (alanine aminotransferase): normal range: 7–41 IU/L; CSF (cerebrospinal fluid) protein: normal range: 15–50 mg/dL; CSF glucose: normal range: 40–70mg/dL; CSF WBC: normal: ≤0.005 cells × 10^3^/L; CSF RBC: normal: 0 PCR: polymerase chain reaction

	Case 1	Case 2	Case 3	Case 4	Case 5
CSF Appearance	Clear	Clear	Clear	Clear	Clear
CSF Protein (mg/dL)	31	55	63	91	43
CSF Glucose (mg/dL)	74	101	70	71	92
CSF WBC (x 10^3^/UL)	0.054	0.100	0.189	0.398	0.538
CSF RBC (x 10^6^/UL)	0	0	0	0.003	0
CSF Lymphocyte count (%)	84%	95%	80%	66%	95%
CSF Gram Stain	No organism seen	No organism seen	No organism seen	No organism seen	No organism seen
Bio-Fire Film Array Panel	Enterovirus PCR detected	Enterovirus PCR detected	Enterovirus PCR detected	Enterovirus PCR detected	Enterovirus PCR detected
Random serum Glucose (mg/dL)	126	110	86	112	165
Hb (g/dL)	8.7	15.0	13.5	14.5	15.1
WBC (x 10^9^/L)	7.6	11.4	8.2	9.7	8.5
Platelets (x 10^9^/L)	207	253	180	225	230
ALT (IU/L)	19	51	Not available	Not available	37
AST (IU/L)	19	33	Not available	Not available	Not available

Empirical IV antibiotics that were started initially were discontinued. Symptomatic treatment was given along with IV hydration. The patient remained afebrile and stable throughout admission. The headache improved with oral analgesics and he was discharged home on the third day. On a follow-up visit to the clinic two weeks later, his symptoms had completely resolved and he was able to resume daily routine activities. 

Case 2

An 18-year-old male, with no prior co-morbidities presented to the ED in September 2020 with a one-day history of low-grade fever, headache, and two episodes of vomiting. Fever was undocumented, not associated with chills or rigors, and relieved intermittently with antipyretics. The headache had a sudden onset, was continuous, moderate to severe in intensity, and localized at the frontal and bilateral temporal regions. There was no history of blurring of vision, seizures, loss of consciousness, altered mental status, cough, diarrhea, or skin rash. He denied eating or drinking anything from outside. On arrival in the ED, he was awake, alert, and was well-oriented to time, place, and person. He was vitally stable and afebrile at that time. Pupils were bilaterally equal, round, and reactive to light. Fundoscopic examination was normal. His neck was stiff. There was no focal neurologic deficit. The rest of the systemic examination was unremarkable. He was admitted to neurology service for acute meningitis and was started on IV hydration and empirical antibiotic coverage for meningitis. MRI brain with contrast and MR venogram were done but were unremarkable. This was followed by a lumbar puncture that elevated the total leukocyte count with 95% lymphocytes. BFM of the CSF detected EV PCR. Results of other laboratory investigations are as shown in Table 1. The diagnosis of enteroviral meningitis was confirmed and antibiotics were stopped, while IV hydration and anti-emetics were continued. He remained afebrile and was sent home the next day. On follow-up visit at the neurology clinic in the following week, he was completely symptom-free.

Case 3

A 35-year-old male with no prior co-morbidities presented to the ED in September 2020, with a two-day history of headache and nausea. Headache was described to have a sudden onset, was constant, bi-frontal, throbbing, severe in intensity with no radiation. There was associated nausea but no vomiting, photophobia, or phonophobia. The pain was relieved only temporarily with IV analgesics. He denied any past history of headaches. One week prior to this presentation, he had an undocumented fever and watery diarrhea that resolved with antipyretics and oral hydration in two days and the patient was able to continue his daily activities. On physical examination in the ED, he was fully conscious, well-oriented but ill-looking. He was afebrile and vitally stable. Neck stiffness was present and Kernig’s sign was positive. His pupils were bilaterally equal, round and reactive to light. Fundoscopic examination was unremarkable. The cranial nerves were intact. Motor and sensory systems were normal. Other systemic examinations were unremarkable. A clinical diagnosis of meningitis was made and he was investigated accordingly. MRI brain with contrast was unremarkable. CSF examination showed elevated total leukocyte count with a lymphocytic predominance of 80%. EV PCR was positive on BFM panel of the CSF. Results of other investigations are as shown in Table 1. He was admitted to neurology service and symptomatic treatment was given. Headache improved with analgesics and IV hydration. The patient remained clinically stable throughout admission and on the third day he was sent home on oral analgesics, to be taken as needed. He visited the neurology clinic a week later and his symptoms had completely resolved by then. 

Case 4

A 19-year-old male, with no prior co-morbidities, presented to the ED in October 2020 with a two-day history of high-grade fever and headache. The fever was documented upto 101 F, was intermittent, relieved with oral antipyretics, and not associated with rigors or chills. The headache had a sudden onset, was generalized, throbbing in nature, severe in intensity, relieved only temporarily with IV analgesics, and caused a hindrance in his daily activities. Two days prior to this he had nausea and loose stools as well, which were managed with oral hydration at home. He mentioned that he had eaten dinner at a nearby restaurant the night before the onset of these symptoms. On arrival in the ED, he was awake, alert, and well-oriented to time, place, and person. He was afebrile and vitally stable. Pupils were bilaterally equal and reactive to light. Fundoscopic examination was normal. Neck stiffness was present. Cranial nerve examination, motor, and sensory systems were within normal limits. Other systemic examinations were unremarkable. Baseline workup was done. Dengue antigen was negative and blood smear did not show malarial parasite. With a working diagnosis of meningitis, he was admitted to the neurology special care unit. CT head was suggestive of mild cerebral edema. CSF studies were done, which showed elevated total leukocyte count with a lymphocytic predominance of 66%. EV PCR was detected on BFM panel of the CSF. Results of other relevant investigations are summarized in Table 1. IV hydration was continued and he was treated with analgesics and antipyretics as needed, to which he responded well. He was discharged home a day later to follow up in the neurology clinic in a week. Upon evaluation in the clinic, he was asymptomatic and had recovered completely. 

Case 5

A 21-year-old male, with no prior co-morbidities presented to the ED in October 2020 with a four-day history of low-grade fever and headache. Fever was undocumented, intermittent, not associated with chills or rigors, and relieved by antipyretics. The headache had a sudden onset, was constant, severe in intensity, and localized at the bilateral temporal regions. There was no history of visual disturbance, seizures, loss of consciousness, or altered mental status. There were no associated gastrointestinal complaints and he denied eating anything from outside. On arrival in the ED, he was awake, alert, and was well-oriented to time, place, and person. Blood pressure was 140/78 mmHg, heart rate was 90 beats per minute, and he was afebrile at that time. Pupils were equal and reactive to light. Neck was stiff and powers were equal in all four limbs with flexor plantar responses bilaterally. The rest of the systemic examination was unremarkable. He was admitted to neurology service with a clinical diagnosis of meningitis and was started on IV hydration and antibiotics in meningitic doses. MRI brain with contrast and MR venogram were done that were unremarkable. This was followed by a lumbar puncture that elevated the total leukocyte count with 95% lymphocytes. BFM of the CSF detected EV PCR. Results of other laboratory investigations are as shown in Table 1. The diagnosis of enteroviral meningitis was made and symptomatic treatment was given. Antibiotics were stopped, while IV hydration and analgesics were continued. He remained afebrile and was sent home on the second day. On a follow-up visit at the neurology clinic in the following week, he showed complete recovery and had resumed his routine activities.

## Discussion

We present five consecutive cases of meningitis associated with acute non-polio EV infection managed at the Aga Khan University Hospital, Karachi, Pakistan. They presented during a span of five weeks in the monsoon season between August and October, 2020. EV infection is seasonal and mostly seen in temperate climates (late summer and autumn) but high around the year in tropical and subtropical countries. Inadequate hygiene and poor sanitary conditions encourage the spread of EV infections in the community.

EVs belong to the family of picornavirus and include polioviruses, coxsackieviruses, and echoviruses. They are acquired mainly by fecal-oral contamination and sometimes by respiratory droplets. They can cause a wide range of diseases ranging from mild illnesses to fatal conditions, with CNS infections accounting for over 70% of viral meningitis cases in children and adults. There is a slight predilection for children and a male preponderance [3]. It causes approximately 75,000 cases of viral meningitis annually in the United States [4]. There is a dearth of data regarding enteroviral meningitis in Pakistan. According to a study carried out at a tertiary care center in Karachi, Pakistan, on the outcomes of treated clinical meningitis in the pediatric population, limited viral cultures and antigen testing were done in the CSF due to either unavailability or pricey cost of the same [5]. This serves as a hindrance in isolating the offending agent, hence enabling unnecessary usage of antibiotics. 

Meningitis is the most common CNS manifestation and encephalitis occurs less frequently. It was found responsible for causing encephalitis in 73 (4.6%) of the 1571 patients with encephalitis that were enrolled in the California Encephalitis Project from 1998 to 2005. Patients with enteroviral encephalitis generally present with milder clinical illness with the exception of encephalitis due to EV71 serotype [6]. The onset of symptoms of enteroviral meningitis is characteristically abrupt and typically includes headache, fever, nausea or vomiting, malaise, photophobia, and meningismus. Our cases presented with severe headache in all five patients (100%), fever in all five patients (100%), and diarrhea in two out of five patients (40%). On examination, neck stiffness was present in all of the five patients (100%).

CSF analysis typically shows a viral picture that includes an elevated total leukocyte count with lymphocytic predominance, a modest elevation in CSF protein concentration, which is generally less than 150 mg/dL, and a normal glucose concentration. As shown earlier, the CSF findings of all the cases presented here were typical of viral meningitis with lymphocytic pleocytosis. However, relevant literature suggests that up to two-thirds of patients with enteroviral meningitis may have a polymorphonuclear predominance in the CSF when examined early in the course of the illness [7]. Repeat lumbar puncture after 12 to 24 hours, if performed, generally shows evolution to a lymphocytic predominance [8]. CSF PCR is the gold standard for diagnosis and was positive in all five of our patients. The sensitivity of reverse transcription-PCR (RT-PCR) assay for EVs in CSF was found to be 100% and specificity was 96.2% in comparison with viral cultures [9].

Fortunately we had the facility to test CSF for EV PCR hence the diagnosis was established and antibiotics were discontinued immediately. Outbreaks of enteroviral meningitis have been reported in the developed countries but there is no data as such from Pakistan. Whether this is due to diagnostic limitations or infrequent cases in the region, this question is yet to be answered. During an outbreak of aseptic meningitis in a pediatric population in our neighboring country, India, stool samples were used for the detection of non-polio EV infections [10]. This approach may sometimes serve as an important clue to diagnosis and can be incorporated across hospitals in Pakistan as well. In order to investigate the etiology of meningitis in the Middle East and North Africa (MENA), a retrospective study was conducted in Qatar, which revealed that EV is responsible for 68.7% of Qatar's viral meningitis cases. While it was detected throughout the year, it peaked significantly during the spring season [11]. Similar studies on meningitis cases need to be conducted in Pakistan and the rest of the South East Asian region to evaluate the etiology and manage patients accordingly. 

​​According to Centers for Disease Control and Prevention guidelines, most non-polio EV infections are self-limited and do not require specific therapy [12]. Potential exceptions include acute myocarditis, which can be life-threatening, CNS infection, and rare but potentially fatal infections in neonates and persons with primary immunodeficiency [13]. There are no FDA-approved antiviral therapeutic agents for non-polio EV. IV immunoglobulin and pleconaril have been studied for certain severe EV infections. Pleconaril is an orally administered antiviral agent that inhibits EV replication by a capsid-binding mechanism. It was tested in two placebo-controlled clinical trials and shortened the course of illness compared to placebo recipients, especially in the early disease course; however, this was a modest benefit and the need of an antiviral agent warrants further research [14].

To minimize the risk of infection, preventive measures have been suggested such as washing hands with soap and water for at least 20 seconds, especially after changing diapers or using the toilet, and avoid close contact, such as touching and shaking hands, with symptomatic persons. There are no preventive vaccines available at present [12].

## Conclusions

In all of our cases, the patient had developed clinical signs and symptoms of meningitis and was initially treated with antibiotics but their workup revealed enteroviral meningitis. The disease course was mild, of short duration, and they were managed with symptomatic treatment only. However, clinical manifestations are generally indistinguishable from other causes of acute meningoencephalitis and range from mild to fatal illness; therefore, a rapid diagnosis is important for the appropriate management and infection control. As mentioned earlier, there is a lack of data on aseptic meningitis including enteroviral meningitis in Pakistan, which warrants the need to study and report disease severity, hospital course, and outcomes in such patients. 
